# Homogeneous self-aligned liquid crystals on wrinkled-wall poly(dimethylsiloxane) via localised ion-beam irradiation

**DOI:** 10.1038/srep08641

**Published:** 2015-03-02

**Authors:** Hae-Chang Jeong, Hong-Gyu Park, Ju Hwan Lee, Yoon Ho Jung, Sang Bok Jang, Dae-Shik Seo

**Affiliations:** 1 Information Display Device Laboratory Department of Electrical and Electronic Engineering, Yonsei University, 50 Yonsei-ro, Seodaemun-gu, Seoul 120-749, Republic of Korea

## Abstract

We demonstrate self-aligned liquid crystals (LCs) using a wrinkled-wall polydimethylsiloxane (PDMS) wrinkle structure, which is a key factor to obtain a stable homogeneous alignment state with positive LCs. We constructed the wrinkled walls via localised surface exposure to IB radiation, which passed through a long length localised pattern mask. The creation of the wrinkled wall helped to align the LC molecules homogeneously because the wrinkled wall acted as a guide for the arrangement of positive LC molecules. In addition, we confirmed the stability of the alignment state as the width of the wrinkled wall was changed. Although this wrinkled-wall method is a non-contact method, LC alignment is achieved via an anisotropic topographical guide, which provides the LC molecules with stable homogeneous alignment.

The uniform alignment of liquid crystal (LC) molecules in a single direction has become an important subject of interest and is a core technological requirement for the fabrication of flexible displays. To produce high-quality display devices for commercial applications, a mechanical rubbing process on polyimide (PI) layers has been widely used to produce microgrooves that are capable of aligning LC molecules in a homogeneous or homeotropic orientation^1^. However, the rubbing process has certain disadvantages, including the accumulation of electrostatic charge, debris and local defects caused by contact with the roller^2^. To solve these problems, non-contact methods, such as ion-beam irradiation^3^,^4^ and the photo-alignment method^2^, have been introduced. However, these non-contact methods suffer from alignment instability because of their variable anchoring energy^5^. Recently, to achieve stable alignment using a non-contact method, LC molecules have been self-aligned on an alignment layer using one dimensional (1D) material, which also provides flexibility of the LCs^6^.

Localised patterning is a common interest in surface science. Several experimental methods for anchoring LCs on patterned substrates are known. One of the methods uses a wrinkle structure for the fabrication of arrays of open micro-channel capillaries. However, this method has the limitation that LC molecules are confined in a microstructure^7^. Additionally, chemical patterns are used to anchor liquid crystals (LCs), which has the limitation of being a complicated process that uses various chemical products^8^.

When a soft material is partially treated using focused ion beams (FIBs), a wrinkle structure forms, thereby yielding a height difference between the non-treated and exposed surfaces. Moreover, there may be either an indirect or direct effect on the non-treated regions between the treated regions, which has been the subject of little study. There is a need for additional studies regarding this effect in the context of various applications.

Herein, we propose a method for the self-alignment of positive LC molecules via a localised surface treatment using ion-beam (IB) irradiation that passes through a long localised pattern. By changing the width of the mask pattern and the intensity of the surface-treatment source, we can control the stability of the LC alignment. Under certain source conditions, wrinkled walls cannot form. However, the proper localised exposure of the surface to IB radiation produces a wrinkle structure and results in a height difference between the wrinkles and the non-treated regions. We also observed a smooth surface on the near side of the wrinkle structure, which was an untreated region. When the necessary wrinkle conditions were satisfied, we observed that LC molecules could be stably and homogeneously aligned on the locally treated surface.

## Results

### Untreated PDMS

**Figure 1:**
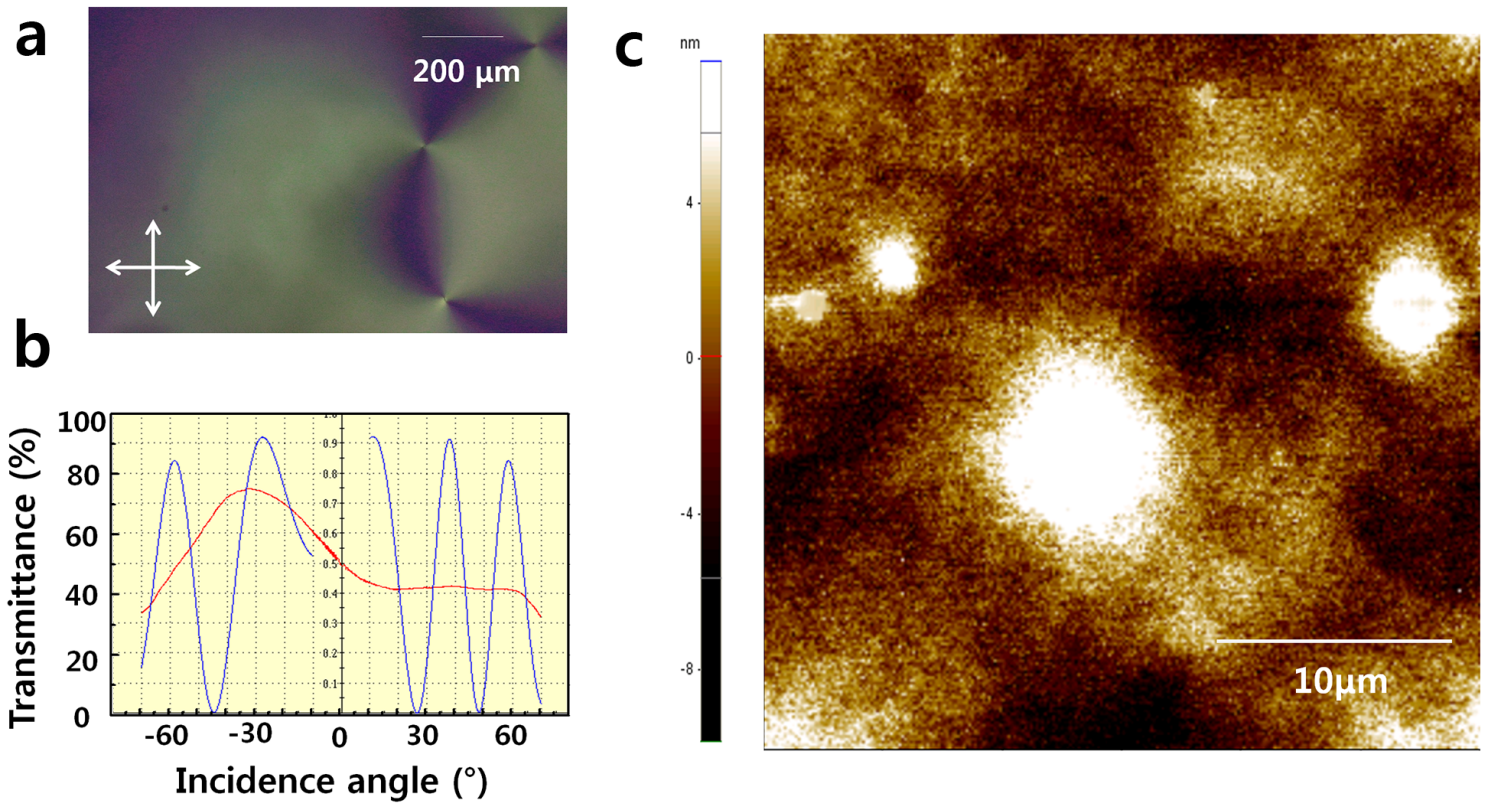
Surface properties of the ‘intrinsic PDMS’ used as an alignment layer. (a) AFM image of the PDMS coated onto ITO-coated glass. (b) POM image of an LC cell that consists of PDMS films. (c) Transmittance measurement performed under latitudinal rotation using a crystal-rotation method. The blue line and the red line represent the simulated and experimental values, respectively.

The alignment state of an LC cell fabricated using polydimethylsiloxane (PDMS) films was investigated via polarising optical microscopy (POM) images, as shown in Fig. 1a. Specific optical patterns, which are known as the schlieren texture of the nematic phase, were observed. In Fig. 1a, there are 3 singularity points, each of which is associated with two black ‘brushes’, which extend in different directions for each singularity. The formation of the schlieren texture is attributed to the non-anisotropic nature of the method, which is in contrast to the rubbing method or ion-beam treatment^1^,^3^,^4^. Therefore, an untreated PDMS film, referred to here as ‘intrinsic PDMS,’ can allow for various LC orientations. As shown in Fig. 1b, the oscillation of the transmittance was measured by rotating the LC cell (TBA 107, Autronic). The blue line represents the simulated curve, whereas the red line represents the experimental curve. If the measured and simulated curves are identical, then the LC alignment is uniform, and the pre-tilt angles of the LC can be accurately estimated. The large difference between the simulated and experimental curves observed here indicates that the LC molecules on the intrinsic PDMS film took on a random alignment, in accordance with the phenomenon observed in Fig. 1a.

The morphology of the PDMS film was investigated via atomic force microscopy (AFM), as shown in Fig. 1c. The white points in the AFM images represent heights greater than 6 nm. The morphology data of the intrinsic PDMS film extracted from AFM are presented in Table 1. A root mean square (RMS) of 2.916 nm was obtained for the intrinsic PDMS film, consistent with previous findings^9^, and its kurtosis was 3.072. The kurtosis is the sharpness of the surface height distribution and represents a Gaussian-like surface centred at 3. A value of the kurtosis greater than 3 indicates that the surface is bumpy and referred to as ‘leptokurtic.’ The many high peaks and low valleys on such a surface prevent the homogeneous alignment of LC molecules.

### Investigation of the alignment state using a wrinkled wall with a width of 50 μm

**Figure 2:**
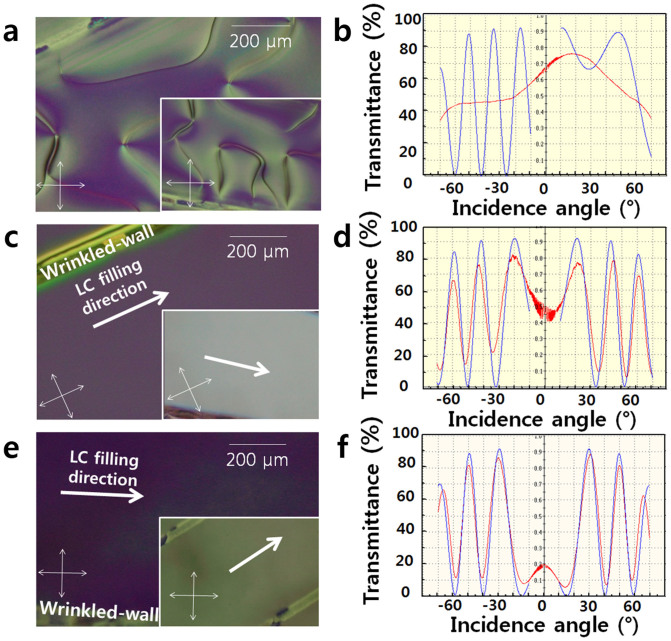
POM images of LC cells composed of narrow-patterned PDMS films fabricated at IB powers of (a) 600 eV, (c) 1200 eV and (e) 2400 eV. The bottom-right POM image insets in each POM image were prepared to demonstrate the homogeneous alignment state of each sample rotated by 45° relative to the polarisers. (b, d and f) Transmittances measured under latitudinal rotation using a modified crystal-rotation method for narrow-patterned PDMS fabricated at 600 eV, 1200 eV and 2400 eV, respectively.

We fabricated the wrinkled wall using a localised surface treatment and the wrinkled wall created on the PDMS used as an alignment layer to align the LC molecules. To irradiate a localised surface via IB, we used a mask pattern with a narrow width and long length, with dimensions of 50 μm by 2 cm. The LC cells with wrinkled walls were observed via POM, and the pre-tilt angle was measured (Fig. 2). A schlieren texture was observed at an IB power of 600 eV (Fig. 2a), which highlighted the importance of the presence of the wrinkled walls. On the films without wrinkled walls, the LC molecules did not demonstrate a preferred direction, thereby resulting in a random LC alignment. The LC molecules injected along the lengthwise direction of the wrinkled wall, which is the same as the lengthwise direction of the mask pattern, exhibited good homogeneous alignment. The direction of the LC alignment was determined by the lengthwise direction of the wrinkled wall. The transmittance of the LC alignment can confirm the LC molecule alignment direction. Well-aligned LCs were observed at IB powers of 1200 eV and 2400 eV, and the wrinkled wall was positioned parallel to one axis of the polarisers. The samples were then rotated by 45° relative to the polarisers (see the insets of Fig. 2c and 2e), and the transmittance was maximised. Because the LCs are a birefringent material, when the aligned positive LCs are placed between the intersectional polarisers, the direction of the LC alignment determines the light transmittance. When the positive LCs are aligned along one direction of the intersectional polariser, the amount light that passed through the LCs was minimised, and we observe a dark image, whereas when the positive LCs are rotated 45° relative to the polariser, the transmittance is maximised. As shown above, the aforementioned transmittance property of the well-aligned positive LCs was observed along the lengthwise direction of the wrinkled wall. Therefore, the direction of positive LC alignment was parallel to the direction of the lengthwise wrinkled wall. The optical characteristics shown in Fig. 2c and 2e are attributed to the direction of LC alignment being parallel to the lengthwise wrinkled wall, which is also the same direction as the filling direction. The data from the rotation method that was used to measure the pre-tilt angle of the LC shown in Fig. 2d and f support this stable homogeneous alignment state. The values of the pre-tilt angle of each sample at 1200 eV and at 2400 eV were 0.192° and 0.258°, respectively. These values were determined with high reliability because the error rates of the pre-tilt angle were 0.03 and 0.022 at 1200 eV and at 2400 eV, respectively. By contrast, filling along the widthwise direction of the wrinkled wall did not exhibit a stable alignment state and exhibited oily streaks along the filling direction (see Supplementary Fig. S1 online). This different phenomenon will be referred to later in this paper. Consequently, stable homogeneous alignment was observed when the filling direction was parallel to the lengthwise direction of the wrinkled wall. In addition, the wrinkled-wall LC alignment ensured a high thermal budget of 150° (see Supplementary Fig. S2 online). The thermal stability temperature of the wrinkled wall is sufficient for LC displays.

### Morphological analysis of the wrinkled walls with widths of 50 μm

**Figure 3:**
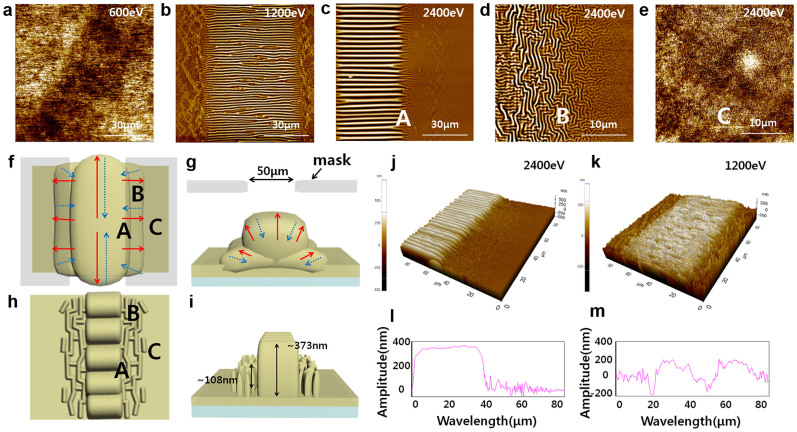
Surface properties of narrow-patterned PDMS used as an alignment layer. The pattern width was 50 μm. AFM images of narrow-patterned PDMS films fabricated at IB powers of (a) 600 eV, (b) 1200 eV, and (c, d and e) 2400 eV. (c) The left side of the PDMS irradiated with the IB and the right side of the PDMS not irradiated with the IB in one sample denoted as region A are shown. (d) A region 15 μm away from the edge of the IB-irradiated site. (e) Centre region between IB-irradiated sites, where an LC is aligned. (f, g, h and i) Schematic depiction of the mechanism for the formation of narrow-patterned PDMS films through IB treatment. (f and g) Top side of narrow-patterned PDMS. The red lines indicate the directions of expansion of the PDMS caused by the heat of the IB irradiation. The blue lines indicate the directions of cooling-induced contraction of the PDMS. (g and i) Lateral side of narrow-patterned PDMS. (j) 3D image corresponding to (c). (k) 3D image corresponding to (b). (l and m) Cross-line profiles of patterned PDMS treated with IB irradiation at 2400 eV and 1200 eV, respectively.

The morphologies of the wrinkled walls created on the PDMS were investigated via AFM, as shown in Fig. 3. The IB irradiation created wrinkle structures on PDMS. When the IB reaches the surface of the PDMS, a stiff skin layer was generated, thereby resulting in the expansion of the PDMS as a result of the energy of the IB exposure. As time passed, this stiff skin layer caused the PDMS to contract. This process resulted in the formation of a wrinkle structure^10^.

The IB contacted the surface exposed by the patterning mask, and a localised wrinkle structure formed as a result. When the IB power was less than a critical wrinkle-formation threshold, the surface of the PDMS remained flat, and wrinkles did not appear^11^. Above the critical wrinkle-formation threshold, the amplitude and wavelength of the wrinkles increased as the IB power increased. Because of the narrow width of the mask pattern used in this study, well-aligned wrinkles formed along the widthwise direction of the mask. The relative difference in stress that caused the wrinkle structure to form in a parallel alignment is analogous to the strain induced on a substrate by stretching^12^,^13^ (Fig. 3f).

Wrinkles appeared not only on the area of the PDMS exposed to the IB (the left-hand side of A) but also on the adjacent boundary (region B and the right-hand side of region A). The energy generated by the IB exposure was transferred outward from the edge of the exposed region (Fig. 3f). This transferred energy caused the PDMS in region B to expand. When the contraction process occurred, in the direction opposite to that of the spreading caused by the transfer of energy, compressive strain was generated along the widthwise direction of the mask. Consequently, a wrinkle structure was formed along the direction of compressive strain, and an anisotropic wrinkle structure was formed along the lengthwise direction of the mask (B in Fig. 3f and Fig. 3h)^12^,^13^. The creation of the wrinkle structure as a result of this swelling process^10^ is referred to as the ‘near-site effect.’ Wrinkle structures induced in the IB-exposure region and the ‘near-site effect’ region act as ‘wrinkled walls.’

To confirm the existence of these ‘wrinkled walls,’ 3D images and line profiles were acquired via AFM, as shown in Fig. 3j–m. The white points in Fig. 3j and Fig. 3k represent heights greater than 375 nm and 200 nm, respectively. The line profiles indicate the existence of wrinkles on the boundary region and the heights of the ‘wrinkled walls’ (Fig. 3l and Fig. 3m). The height of the wrinkled walls increased as the IB power intensity was increased. Subjecting a surface to IB irradiation increased the compressive stress on that surface^14^, this phenomenon caused the characteristic height differences in the wrinkle structure. The wrinkled walls in the region exposed to the IB and the boundary region had average heights of 373 nm and 108 nm, respectively, at an IB power of 2400 eV and average heights of 203 nm and 57 nm at 1200 eV.

Region C represents the remainder of the PDMS that was not exposed to the IB. The morphology of region C was smooth compared with that of the intrinsic PDMS. The root mean square (RMS) of region C was 2.45 nm, and its kurtosis value was less than 3, as presented in Table 1, thereby indicating that the surface was flat and ‘platykurtic.’ The change in morphology between the intrinsic PDMS (Fig. 1c) and region C (Fig. 3h) may have been induced by the creation of the wrinkled walls. Although neither surface was irradiated with the IB, subjecting a localised surface region to IB irradiation increases the compressive stress on that region^14^, which may also result in the application of a transversal stress to the non-treated region between the treated regions, thereby creating the smoother morphology compared with that of intrinsic PDMS that was observed in region C (Fig. 3h).

### The influence of the width of the wrinkled wall and morphology analysis

**Figure 4:**
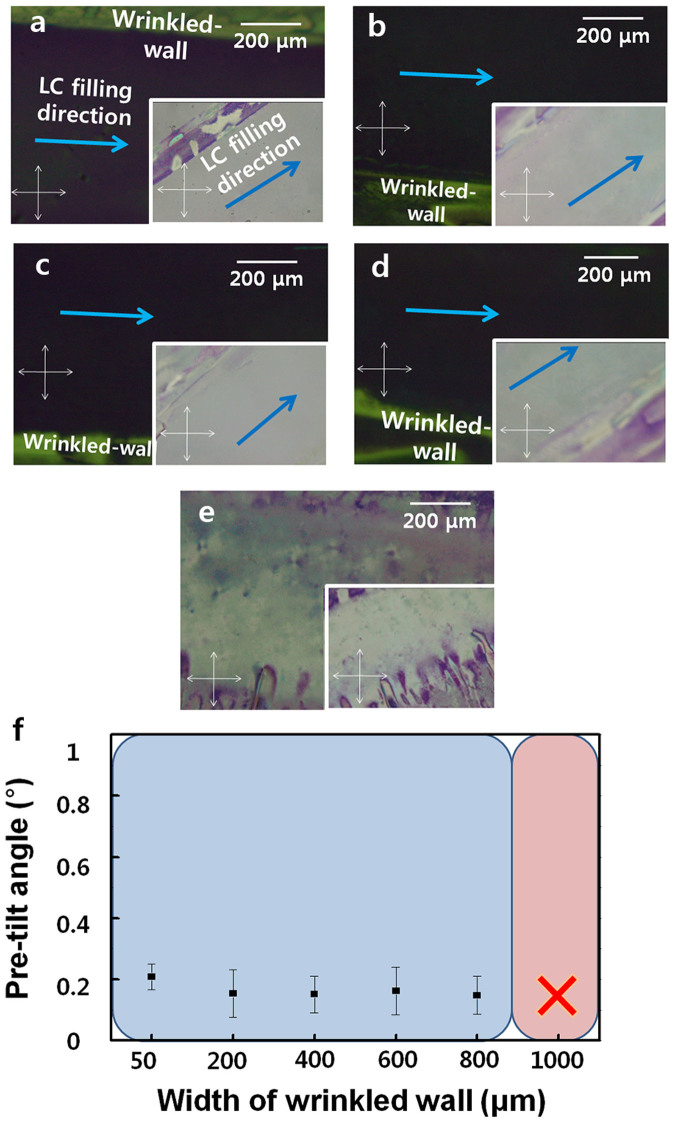
POM images of PDMS with various wrinkled-wall widths. The pattern widths are (a) 200 μm, (b) 400 μm, (c) 600 μm, (d) 800 μm and (e) 1 mm. The bottom-right POM image insets in each POM image were prepared to demonstrate the homogeneous alignment state of each sample rotated by 45° relative to the polarisers. (f) The pre-tilt angle of the wrinkled wall as a function of the width variation.

To examine the influence of the changing width of the wrinkled wall on the alignment of the LC molecules, we changed the width of the mask and fabricated the LC cells with patterned wrinkled walls with various widths. Stable homogeneously aligned LCs were observed on PDMS films with wrinkled walls (Fig. 4) in which the width was less than 800 μm, with clear pre-tilt angles (see Supplementary Fig. S3 online). However, the wrinkled wall with a width of 1 mm exhibited a random alignment (Fig. 4e). When rotating a sample by 45 degrees, dark images change into white images; thus, these results indicate that the LC molecules were aligned homogeneously along the lengthwise direction of the wrinkled wall. The graph yielded by the crystal-rotation method shows that the experimental results and simulated data are perfectly consistent (see Supplementary Fig. S3 online). The pre-tilt angles of each sample for widths of 200 μm, 400 μm, 600 μm and 800 μm averaged 0.15, 0.15, 0.16 and 0.14, respectively (Fig. 4f). These data support the POM analysis. The calculated pre-tilt angles of LC molecules on PDMS with a wrinkled-wall width of less than 800 μm were under 0.2° with high reliability, thereby demonstrating an acquirement of stable low pre-tilt angles regardless of the wrinkled-wall width. This property is useful for touch-based devices with fast-recovery of LC domains because of their low variation and for obtaining a wide viewing angle and avoiding the light-leakage problem. However, for the width of 1 mm, we did not extract the pre-tilt data because of the random alignment state confirmed by the POM images.

The morphologies of the wrinkled wall as the width changed exhibited a similar trend to that of the wrinkled wall with a width of 50 μm (see Supplementary Fig. S4 online). On the region treated by IB, an anisotropic wrinkle structure was formed because widthwise stress was induced by the mask. In addition, the anisotropic wrinkle structure on the ‘near-site effect’ region was formed along the lengthwise direction of the mask, as for a width of 50 μm. The lateral sizes of the ‘near-site effect’ region were enlarged as the mask width was increased because of the increase in absorption energy caused by enlargement of the treated area.

**Figure 5:**
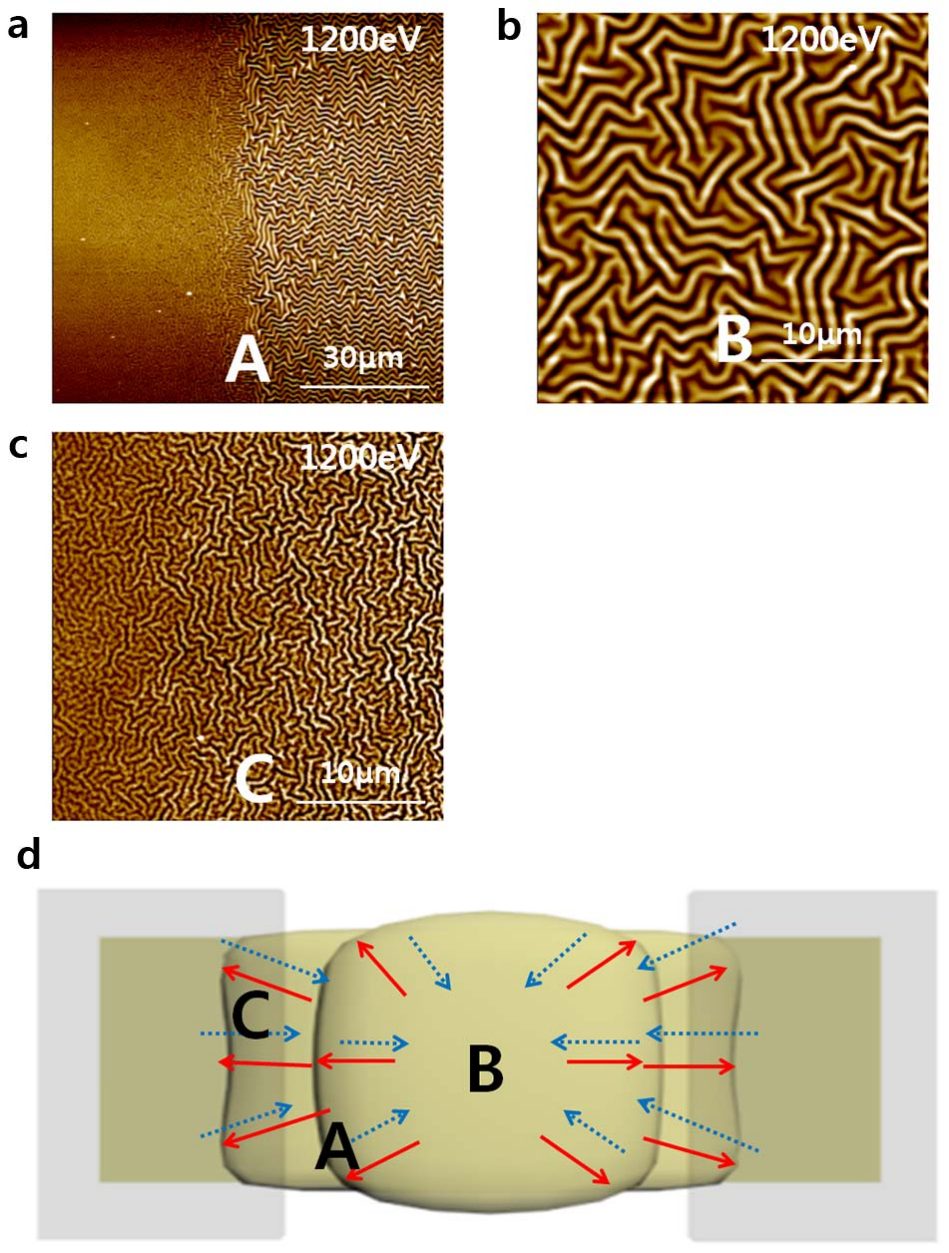
AFM images of wide-patterned PDMS films fabricated at IB powers of (a, b, and c) 1200 eV. The pattern width was 1 mm. (a) Interface between a region exposed to the IB irradiation (left-hand side) and a region not exposed to the IB irradiation. (b) Centre of a region exposed to the IB irradiation. (c) Region 80 μm from the edge of the exposure site. (d) Schematic illustration of the mechanism for the formation of wide-patterned PDMS films through IB treatment. The regions denoted by A, B, and C in (d) represent the regions shown in (a), (b) and (c), respectively.

The swelling mechanism described above could also be applied in the interpretation of this experiment, but the wrinkle formation was different than that observed in the previous trial. It has been demonstrated that adjusting the level of relative strain between planar directions can lead to the creation of a sequential structure from 1D to random, in which a structural change point exists^12^. As the mask width increased by 1 mm, a random labyrinthine structure was observed, as shown in Fig. 5b and in the B region of Fig. 5d. Enlargement of the mask-pattern width may produce the same effect as that of biaxial stress applied to the surface and passing through the structural change points, thereby resulting in the formation of a random labyrinthine structure, similar to a sequential process adjusting the level of the relative strain. Furthermore, the swelling process might not remain equibiaxial across the entire surface^13^. Although the surface was identically exposed to the IB, the swelling process of the surface exposed to the IB induced non-equibiaxial stress, thereby resulting in the formation of a different morphology at the centre (region B in Fig. 5d) from the morphology that formed toward the edge (the right-hand side of Fig. 5a). Ordered herringbone patterns arose near the edges, approximately 50 μm from the right-hand side of Fig. 5a, whereas region B exhibited a random structure.

A random pattern was observed 80 μm from the edge of the exposed site in region C in Fig. 5d (corresponding to Fig. 5c). The aforementioned ‘near-site effect’ occurred within region C. However, the energy generated by the IB exposure was carried far from the edges because of the increased exposure surface, thus propagating the swelling process farther toward the non-treated region C. As the coverage of the swelling process in region C increased, the compressive stress on the ‘near-site effect’ region no longer induce the anisotropic formation, as in the case of the treated region. Enlargement of the coverage of the swelling process caused the difference in the relative stress in all directions, which have led to the formation of the random wrinkle structure observed in region C in Fig. 5e (Fig. 5c).

## Discussion

**Figure 6:**
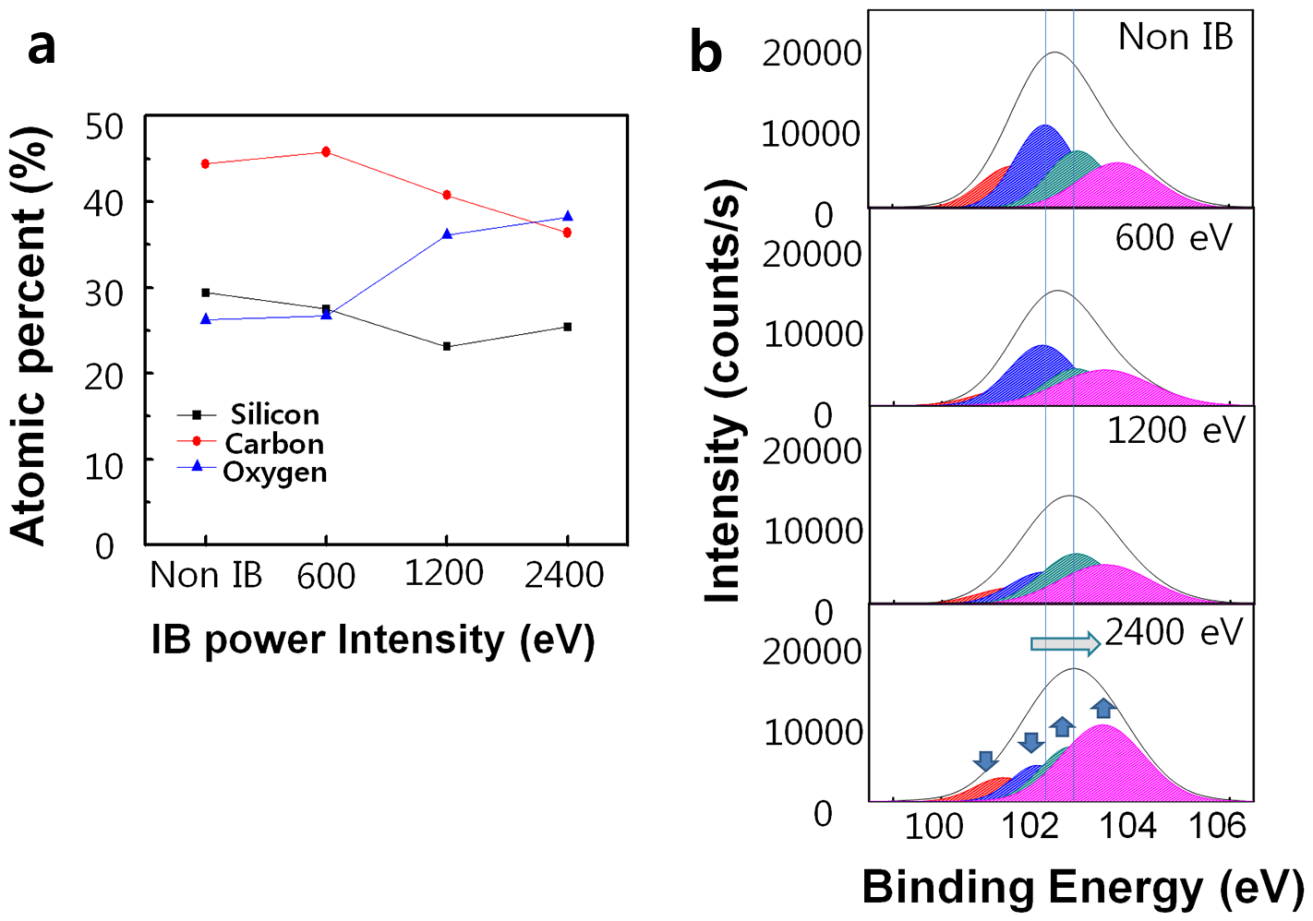
XPS analysis of an IB-treated region in which the wrinkle structure was formed. (a) Variation in the atomic concentrations of Si, O, and C as a function of IB power intensity. (b) Si 2p core-level XPS spectra of the surface according to the IB power intensity.

After selectively irradiating the PDMS via the mask pattern, IB exposure transformed the PDMS surface to a silicon oxide layer and created a wrinkled wall. The mechanism of ion beam-induced transformation is based on the wrinkled hard skin^11^. IB irradiation breaks the polymer chain and bonds with oxygen atoms that cause a stiff thin film. A stiff thin film increased the compressive stress on that surface^14^, leading to wrinkle formation, i.e., a wrinkled wall. X-ray photoelectron spectroscopy (XPS) confirmed the transformation of the PDMS surface into an oxidation layer. First, changes in atomic concentration were caused by IB irradiation (Fig. 6a). As the surface was irradiated by IB and the power of the IB was increased, the amount of oxygen increased from 25% to 37% and the carbon content decreased from 45% to 37%. This result is attributed to oxidation of the PDMS skin layer by IB irradiation.

The chemical structure of PDMS is CH_3_[Si(CH_3_)_2_O]_n_Si(CH_3_)_3_, which consists of repeated monomer units of Si(CH_3_)_2_O. To confirm the effect of IB irradiation in detail, we analysed the binding energy of the Si 2p peak of the PDMS, which is related to silicon (Si) and oxygen (O) bonding (Fig. 6b). The four component sub-peaks, which represent Si-O bonding, are (-O)_1_: [(CH_3_)_3_SiO_1/2_], (-O)_2_: [(CH_3_)_2_SiO_2/2_], (-O)_3_: [(CH_3_)SiO_3/2_], and (-O)_4_: [SiO_4/2_]. (-O)_1_ is centred at 101.5 eV, (-O)_2_ is centred at 102.1 eV, (-O)_3_ is centred at 102.8 eV, and (-O)_4_ is centred at 103.4 eV^15^. Non-irradiated PDMS has a higher (-O)_1_ and (-O)_2_ component. After IB irradiation, the (-O)_1_ and (-O)_2_ components were reduced, whereas the (-O)_3_ and (-O)_4_ was increased, as shown in Fig. 6b and Supplementary Table S1 online. IB irradiation led to a higher (-O)_3_ and (-O)_4_ component. This component change in the Si 2p peak induced an increased binding energy for the Si 2p peak. Because atoms of a higher positive oxidation state exhibit a higher binding energy, the right shift of the Si 2p peak indicates that the IB irradiation transformed the PDMS to an oxidised state. At higher IB energy, more oxygen atoms bonded with each Si atom. This result corresponds with the increasing trend of oxygen content that is evident in Fig. 6a. The wrinkled walls in the region exposed to the IB irradiation and the boundary region had average heights of 373 nm and 108 nm, respectively, at an IB power of 2400 eV and average heights of 203 nm and 57 nm at 1200 eV. This result is attributed to the higher oxidation observed through the aforementioned XPS analysis. Therefore, the IB irradiation led to a stiff skin layer through the oxidation process and increased the compressive stress^14^, thereby altering the degree of the oxidation state and creating a wrinkle structure, which is a wrinkled wall.

**Figure 7:**
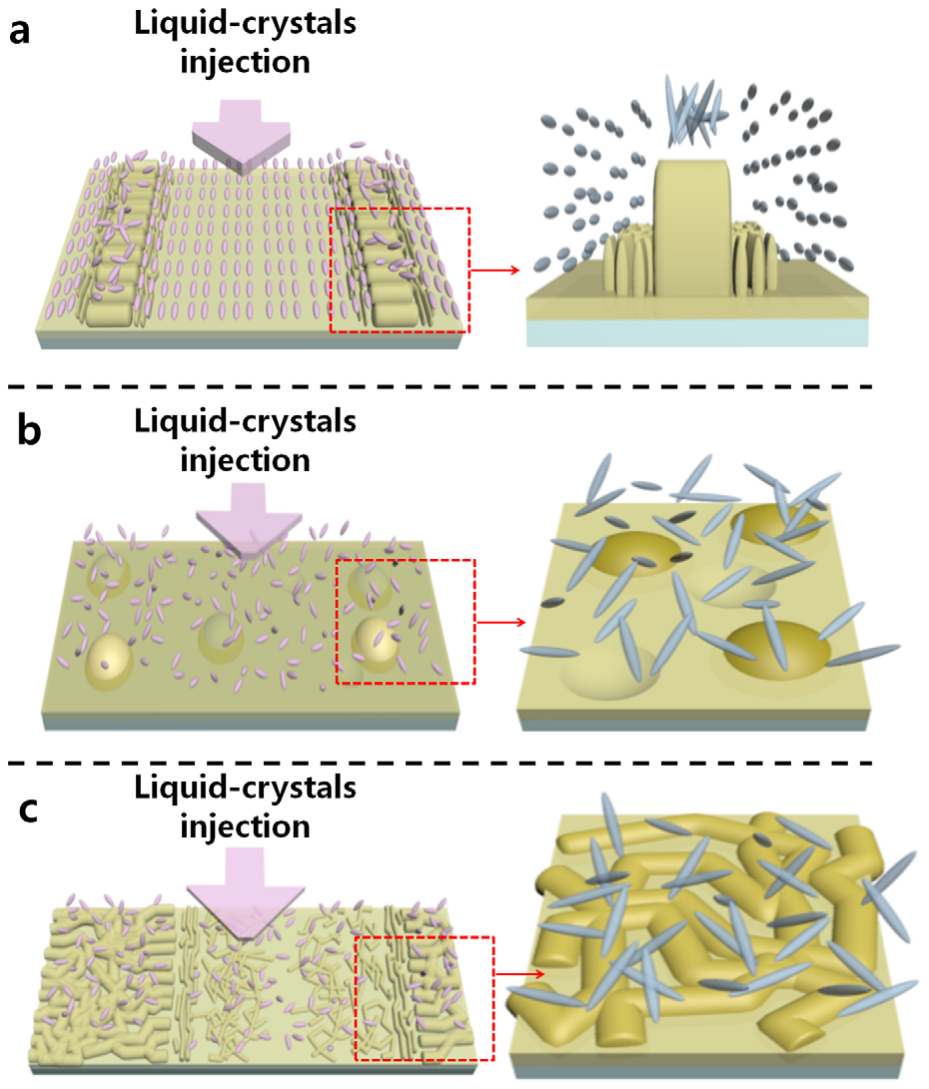
A schematic diagram of the mechanism of LC alignment. (a) On the PDMS regions between sites treated with an IB passing through a narrow pattern under 800 μm (b) On the non-treated PDMS. (c) On the PDMS regions between sites treated with an IB passing through a wide pattern.

A possible homogeneous self-alignment mechanism that involves ‘wrinkled walls’ is proposed in Fig. 7a. The mechanism of conventional rubbing-induced LC alignment remains uncertain. One of the rubbing mechanisms is that periodic microgrooves force the LC molecules to align along the rubbing direction because of topographical properties^16^. A geometric restriction induced by microgrooves between surface and LC molecules acted as a guide for the arrangement of LC molecules, thereby aligning the LC molecules in a single direction. Therefore, it is important to anchor LC molecules in a single direction with a method of inducing anisotropic LC directors. This problem can be solved as inducing the geometric restriction via ‘wrinkled walls’. It has been demonstrated that 1D material arrays such as nanowires, which have a geometry similar to that of LC molecules, can be produced via ‘nano-logging’^17^,^18^,^19^, which uses barriers as a guide for the arrangement of the 1D material. Localised exposure via a mask pattern formed wrinkled walls on the PDMS along the lengthwise direction of the mask. A well was formed between each pair of wrinkled walls. Similar to the nano-logging method, the positive LC molecules were injected between each pair of wrinkled walls via the capillary method along the lengthwise direction of the wrinkled wall. The LC molecules then became arranged along the lengthwise wrinkled-wall direction, as is caused by the barriers used in nano-logging. The LC molecules, which were affected by the wrinkled walls, forced their neighbouring LC molecules to orient in the same direction. This allowed a stable homogeneous LC alignment to propagate into the bulk as a result of the elastic nature of the LC director field^20^. As a result, the wrinkled wall acted as a support for this homogeneous alignment. Simultaneously, the surfaces between wrinkled walls may have contributed by forming an even floor that could maintain the stable homogeneous alignment. Over the IB-exposed regions in which an anisotropic wrinkle structure formed, changes in the chemical composition of the surface affected the LC molecules. From the result of the XPS analysis shown in Fig. 6, the IB-exposed region had higher surface oxidation. The increase in the portion of O_3_ and O_4_ generated the oxidation state of the surface and eliminated delocalised electrons^21^. The removal of the delocalised electrons on the surface decreased the dipole polarisability, thereby inducing a weak van der Waals interaction between the LC molecules and surface. The weak interaction between the surface and LC molecules caused the LC molecules to not be anchored on the surface, which may have interrupted the orientation.

When the LC molecules were injected along the widthwise direction of the wrinkled wall, which is the same direction as that of the anisotropic wrinkle structure on the treated region, it did not exhibit a stable homogeneous alignment for all widths (see Supplementary Fig. S1 online). Although an anisotropic structure was formed on the treated region longer, the aforementioned weak interaction of the surface and LC molecules induced by the surface reformation via IB did not anchor the LC molecules on the surface in a preferred direction, and the structure was not perfectly ordered; consequently, a random alignment was induced^21^. This result strengthens the aforementioned assertion regarding the interaction between the surface and LC molecules. In addition, on the untreated region, the LC molecules that were vertically injected into the wrinkled wall might have been hindered from aligning because of the interference of the wrinkled wall, which acted like a speed bump.

In the case of LC molecules on intrinsic PDMS, a random alignment was induced. As previously confirmed, the surface of the intrinsic PDMS is bumpy compared with that of the IB-exposed, patterned PDMS. As mentioned earlier, a bumpy surface prevents LC molecules from aligning homogeneously and stably. The absence of a guide or other method of creating anisotropic LC directors causes the LC molecules on the PDMS to take on a random alignment, as shown in Fig. 7b.

In wrinkled-wall PDMS with a width of 1 mm, random wrinkling was created across the entire area, except at approximately 50 μm from the edge on the treated region, where a partially ordered herringbone pattern was formed. Because the partially ordered herringbone structure was not uniformly aligned, it was difficult to stably anchor LC molecules oriented in a single direction in this region. Moreover, most regions possessed a randomly wrinkled surface that gave rise to a random orientation of the LC molecules that propagated into the bulk. Therefore, the random wrinkle structure generated a random alignment instead, thereby weakening the effect of the wrinkled walls (Fig. 7c).

In summary, to produce an anisotropic guide for alignment and a smooth surface for stable interaction between LC molecules and the surface, localised IB exposure was applied to a PDMS film through a long, narrow-mask pattern to produce a wrinkled structure. Because of the height difference between the wrinkled structure and the non-treated regions, wrinkled walls formed, which acted as guides for alignment. In addition, the non-treated region between the wrinkled walls was observed to have a smooth, flat surface. Forming a wrinkled wall caused the application of a transversal stress to the non-treated region between the treated regions. Consequently, this combination of smooth surfaces and wrinkled walls created wells filled with positive LC molecules in a process similar to ‘nano-logging.’ This method stably anchored the positive LC molecules. Despite the use of a non-contact treatment, the LC molecules could be as stably aligned as can be achieved using a physical approach, such as the rubbing process. Moreover, the ability to apply this technique at a low temperature allows for potential application to flexible LC devices.

## Methods

### Materials & preparation

Polydimethylsiloxane (PDMS; Sylgard-184, Dow Corning was prepared by mixing the elastomer base and curing agent in a volume ratio of 10:1. The mixture was placed in a vacuum box until all trapped air bubbles were removed, and the PDMS was then spin-coated onto an indium-tin-oxide-coated Corning 1737 glass substrate with dimensions of 2 × 2 cm at 3000 rpm for 30 s. The coated glass was preheated at 65°C for 120 min. Commercial positive LC (*Δϵ* = 10.7, Merck) has refractive indices of 1.4756 on the ordinary axis and 1.5702 on the extraordinary axis. There are some square holes in the mask, the length of these holes is 2 cm, and the widths of these holes are 50 μm, 200 μm, 400 μm, 600 μm, 800 μm and 1 mm. The distances between two neighbouring holes are uniformly 1 mm.

### Ion-beam (IB) irradiation

A Duo PI Gatron ion-beam system was used for IB irradiation. The PDMS samples were placed in a high-vacuum chamber under a working pressure of 3 × 10^−4^ Torr and an Ar gas flow rate of less than 1 SCCM. The IB exposure conditions were as follows: a positively charged current density of 1.2 mA/cm^2^ and energy intensities of 600 eV, 1200 eV and 2400 eV.

### Formation of wrinkled walls

The spin-coated PDMS films were irradiated with an IB passing through a patterning mask. After the films underwent the swelling process caused by the selective IB exposure, a wrinkled structure, which is referred to as a wrinkled wall, formed along the lengthwise direction of the mask under certain IB conditions, 1200 eV and 2400 eV.

### Liquid-crystal cell fabrication

The substrates were fabricated in an anti-parallel configuration with a cell gap of 60 μm to observe the pre-tilt angles and alignment conditions, where the anti-parallel direction was defined to lie along the lengthwise direction of the mask. The LC molecules were then injected between each pair of wrinkled walls along the lengthwise direction of the mask.

### Crystal-rotation method

The oscillation of the transmittance was measured by rotating the LC cell via TBA 107 (Autronic). The blue line represents the simulated curve, whereas the red line represents the experimental curve. If the measured and simulated curves are identical, then the LC alignment is uniform, and the pre-tilt angles of the LC can be accurately estimated. Each sample was measured at 10 different points.

### Surface morphology and analysis of the wrinkled wall patterns using AFM

The surface morphology of the wrinkles was observed via atomic force microscopy (AFM; Park Systems, XE-BIO). To confirm the height difference between the wrinkled wall and the untreated region, we quantified the detailed parameters of the wrinkle morphology using line profiles that were deduced from the AFM images; these profiles represented the amplitude and wavelength of the wrinkle structures.

### Chemical composition analysis

The chemical compositions of the IB-exposed PDMS surfaces were analysed via XPS (K-alpha, Thermo VG, U.K.) using a mono-chromatic Al X-ray source (Al Kα line: 1486.6 eV) and a 12-kV, 3-mA power source. The Si, C, and O atoms of the main chemical components of PDMS were analysed and calibrated by comparison with C 1s (284.8 eV).

## Supplementary Material

Supplementary Information SUPPLEMENTARY INFORMATION

## Figures and Tables

**Table 1:** Comparison between the intrinsic PDMS and the untreated region for the regions exposed to an IB passing through a narrow-mask pattern

Condition	RMS (nm)	Kurtosis
Intrinsic PDMS	2.916	3.072
Untreated region of narrow-patterned PDMS	2.45	2.955
