# Supplementary material for: Homogeneous self-aligned liquid crystals on wrinkled-wall poly(dimethylsiloxane) via localised ion-beam irradiation
Source: Sci Rep. 2015 Mar 2;5:8641. doi: 10.1038/srep08641 (PMC4345327; doi:10.1038/srep08641)
Supplement: Supplementary Information [file srep08641-s1.docx]

**SUPPLEMENTARY INFORMATION**

**Homogeneous self-aligned liquid crystals on wrinkled-wall poly(dimethylsiloxane) via localised ion-beam irradiation**

Hae-Chang Jeong^1,2^, Hong-Gyu Park^1,2^, Ju-Hwan Lee^1^, Yoon-Ho Jung^1^, Sang-Bok Jang^1^ and Dae-Shik Seo^1^

*^1^ Information Display Device Laboratory Department of Electrical and Electronic Engineering, Yonsei University, 262 Seongsanno, Seodaemun-gu, Seoul 120-749, Republic of Korea;*

*^2^ Authors contributed equally.*


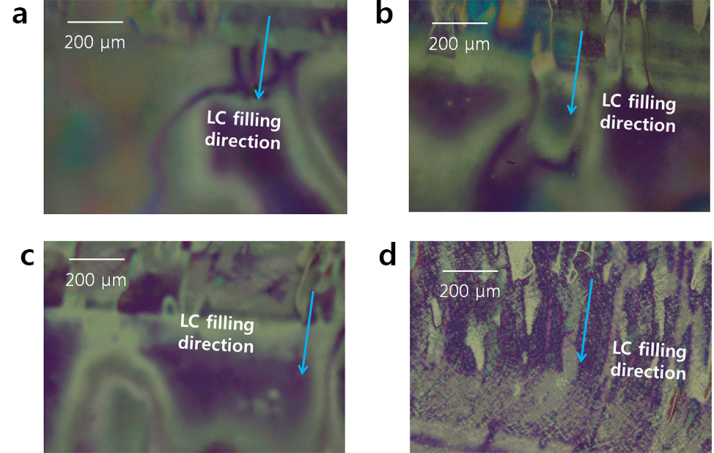


**Figure S1.** POM images of an LC cell when injecting the LC molecules along the widthwise direction of the mask. The mask widths are (a) 50 μm, (b) 200 μm, (c) 800 μm and (d) 1 mm.

To confirm the direction of the LC alignment, an LC cell constructed using wrinkled-wall films was fabricated using different LC filling directions. The LC molecules injected along the widthwise direction of the mask did not exhibit stable homogeneous alignment for all width conditions (Fig. S1). In addition, oily streaks were observed along the LC filling direction. For widths of less than 800 μm, although an anisotropic wrinkle structure was formed on the treated region along the filling direction (Fig. S4), the alignment of LC molecules injected vertically into the wrinkled wall might have been hindered because of the interference of the wrinkled wall, which could have acted as a speed bumper. Over the IB-exposed regions in which an anisotropic wrinkle structure formed, changes in the chemical composition of the surface affected the LC molecules. From the result of XPS analysis (Fig. 6), the IB-exposed region exhibits higher oxidation. The increase in the fraction of O_3_ and O_4_ generated the oxidation state of the surface and eliminated delocalised electrons^1^. Removal of the delocalised electrons on the surface decreased the dipole polarisability, thereby inducing a weak van der Waals interaction between the LC molecules and surface. The weak interaction with the surface caused the LC molecules to not be anchored on the surface. Although an anisotropic wrinkle structure was formed, the LC molecules that were not anchored to the surface prevented the orientation from having a preferred direction, thereby inducing a random alignment state.


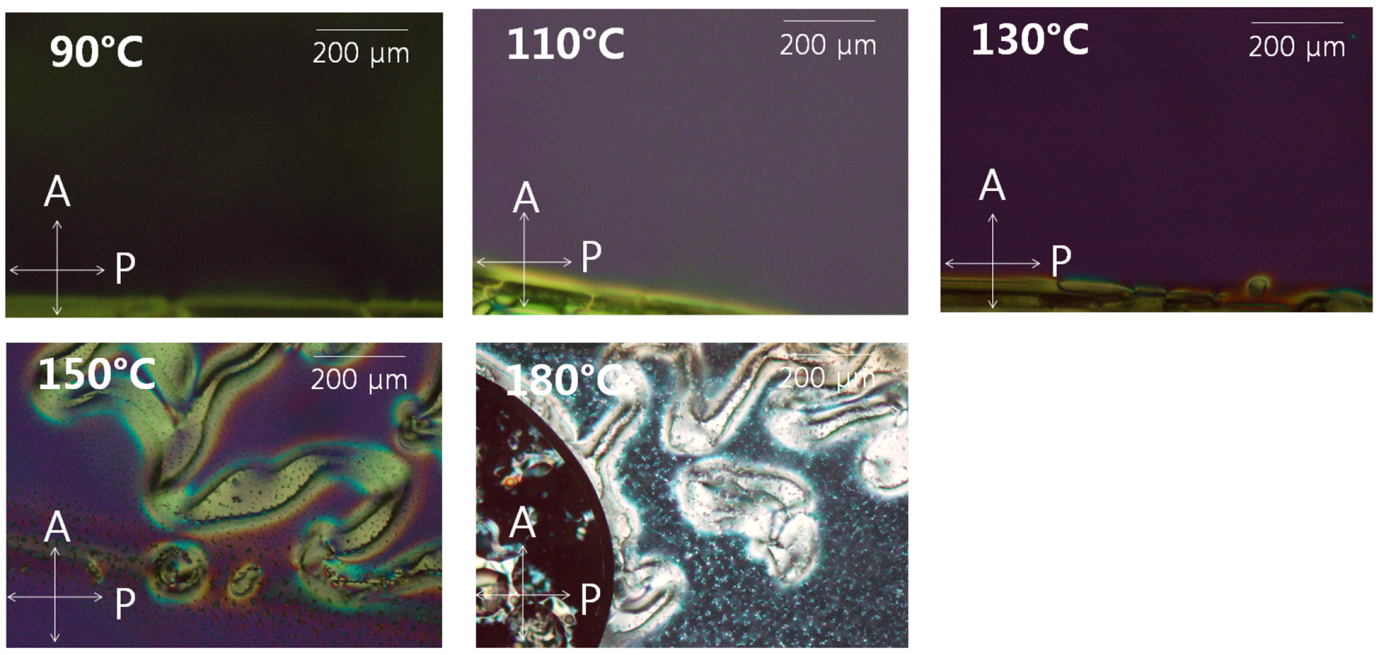


**Figure S2.** POM images for confirmation of the thermal stability of the wrinkled wall with a width of 50 μm at an IB power intensity of 2400 eV.

The main concern about all devices is how the device can endure heat. A high thermal budget is essential for constructing highly durable LCDs. Therefore, thermal-stability testing of LCs is a method for quantitatively evaluating the stability of the LC alignment. The below figures present the thermal stability of the self-aligned LC induced by wrinkled-wall PDMS. The cell fabricated at 2400 eV was cooled gradually after annealing for 10 min at 90, 110, 130, 150 and 170 ◦C. The stable LC alignment remained constant. Above 150 ◦C, the LC alignment was destroyed. The thermal stability temperature of the wrinkled wall is sufficient for LCDs.


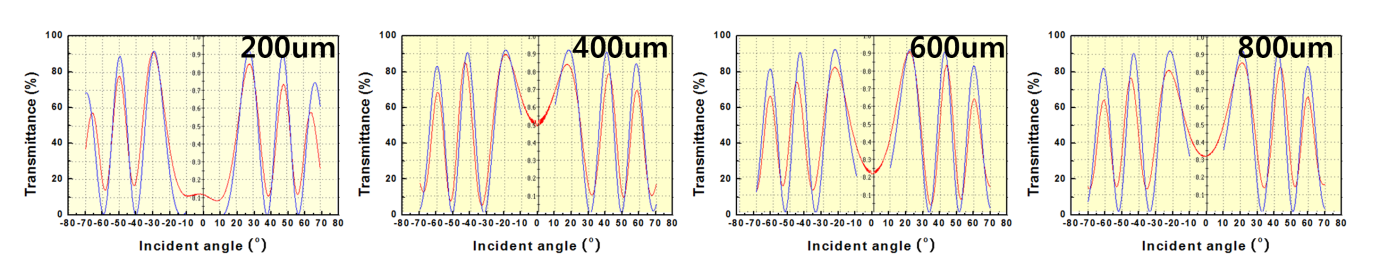


**Figure S3.** Transmittances measured under latitudinal rotation using a modified crystal-rotation method for patterned PDMS; the widths were 200 μm, 400 μm, 600 μm and 800 μm, respectively.

The graphs obtained using the crystal-rotation method indicate that the experimental results are perfectly consistent with the simulated data. The error rates for each pre-tilt graph are 0.019, 0.013, 0.019, and 0.213 at 200 μm, 400 μm, 600 μm and 800 μm, respectively. These values indicate the high reliability of the pre-tilt data. The pre-tilt angles of each sample for widths from 200 µm to 800 µm averaged 0.15, 0.15, 0.16 and 0.14, respectively.


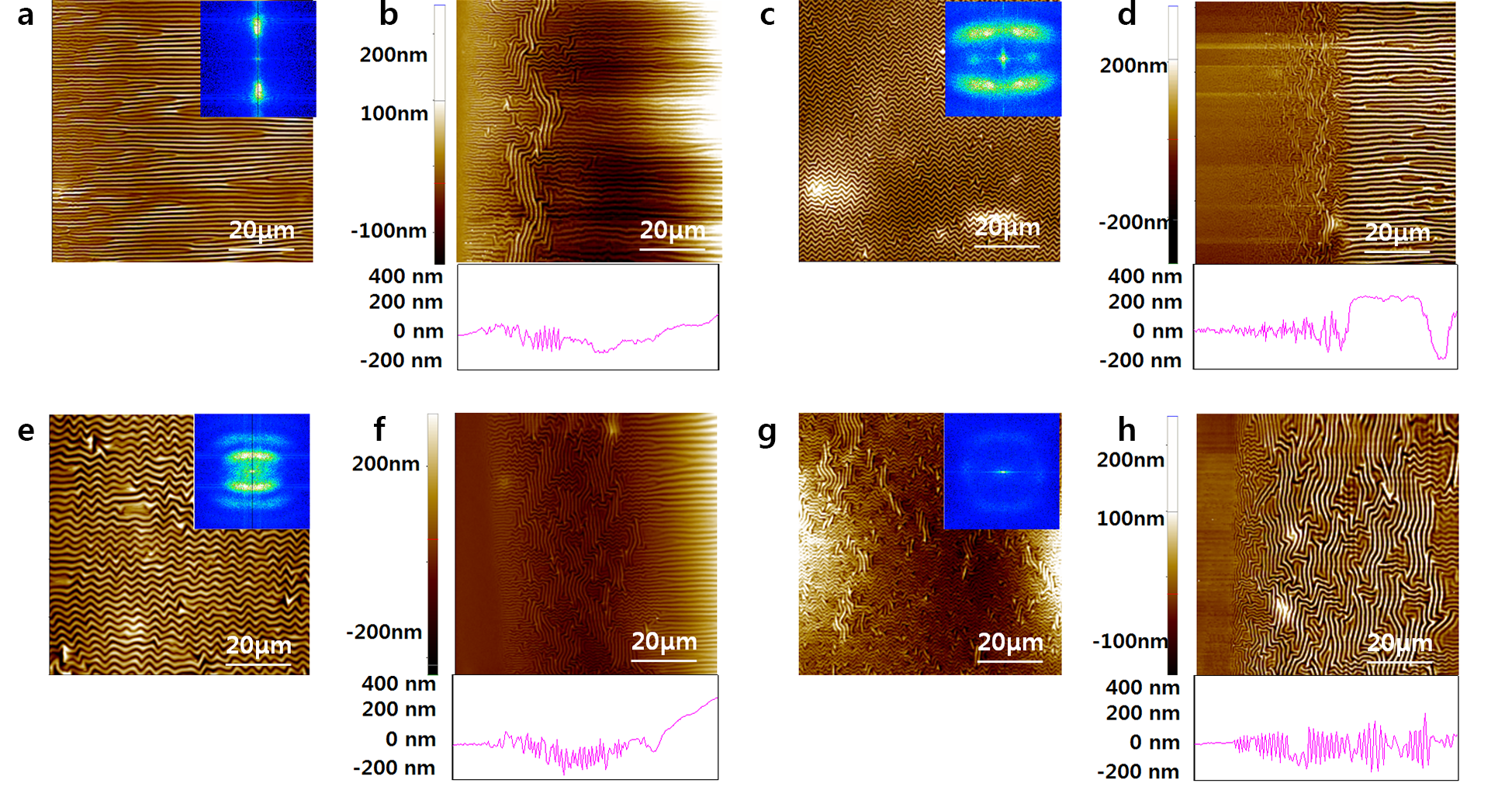


**Figure S4.** Surface properties of the patterned PDMS. The pattern widths were 200 μm in (a) and (b), 400 μm in (c) and (d), 600 μm in (e) and (f), and 800 μm in (g) and (h). (a), (c), (e) and (g) show AFM images of the IB-treated regions, which are wrinkle structures. The top-right inserts for (a), (c), (e) and (g) show fast Fourier transform (FFT) images of the IB-treated region. (b), (d), (f), and (h) show AFM images of the edge of the exposed site. The bottom graphs of (b), (d), (f), and (h) show a line profile along the lateral direction, which demonstrates the ‘near-site effect’.

To analyse the treated region and edge region as the width was varied, the surface morphology for widths of 200 μm, 400 μm, 600 μm and 800 μm was investigated using AFM images (Fig. S4). Independent of the mask width, the region treated by IB exhibited an anisotropic wrinkle structure, although there were slight structural differences (Fig. S4 b, d, f, and h). An anisotropic herringbone structure was observed for a mask width of 400 μm and 600 μm. For a width of 800 μm, some regions exhibited wrinkles that formed perpendicular to the main herringbone structure. There was a relative difference in strain induced by the mask width, and this induced structural differences as the width was changed. However, because the widthwise stress still dominated the wrinkle structure, the anisotropic wrinkle structures was formed until a width of 800 μm was reached^2^. The separated upper side and lower side in the FFT images indicate that the surface was anisotropic. These results demonstrated that a width of less than 800 μm led to the formation of unidirectional wrinkle structures.

The left side of panels in Fig. S4 a, c, e and g show a ‘near-side effect’ region. The ‘near-side effect’ region exhibits an anisotropic wrinkle structure perpendicular to the direction of the wrinkle structure of the IB-treated region. The energy generated by IB exposure was transferred outward from the edge of the exposed region. As for the width of 50 μm, the swelling caused by the energy transfer occurred in a direction perpendicular to the lengthwise direction of the mask. The lateral sizes of the ‘near-side effect’ region were enlarged as the mask width was increased. These phenomena were attributed to the increased region treated by IB. The enlargement of the IB-treated region caused more energy transfer outward and affected the far-edge region, thereby expanding the ‘near-side effect’ region. It is sufficient to demonstrate that compressive stress was generated along the widthwise direction of the mask from the edge, and an anisotropic wrinkle structure was formed along the lengthwise direction of the mask in the ‘near-side effect’ region (Fig. S4 b, d, f and h) ^2^.


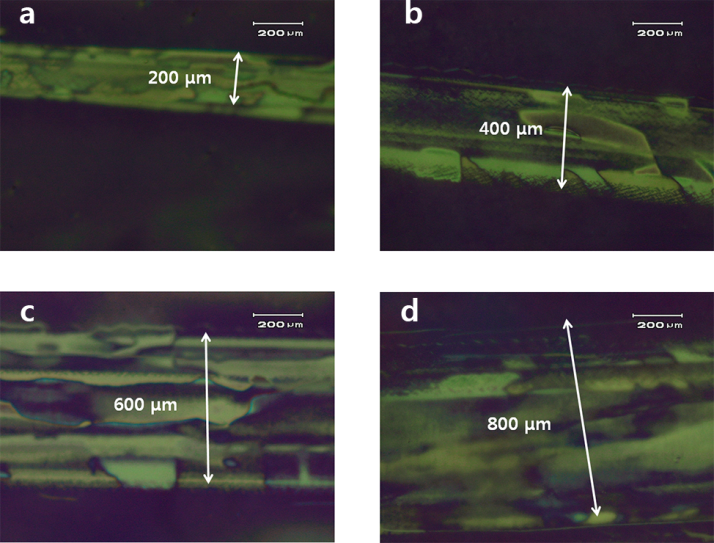


**Figure S5.** POM images of the patterned PDMS. The width of the treated region can be confirmed. On the treated region, the LC molecules were not aligned.

The POM images were used to verify the width of the wrinkled wall induced by localised surface treatment via IB.

**Table S1.** XPS analysis of the Si 2p peak power intensity in terms of the four sub-components related to oxidisation.

|  | O1 | O2 | O3 | O4 |
| --- | --- | --- | --- | --- |
| Non IB | 18.4 | 33.7 | 23.2 | 24.7 |
| 600 eV | 11.8 | 36.8 | 21.2 | 30.2 |
| 1200 eV | 11.6 | 20.1 | 33.9 | 34.4 |
| 2400 eV | 10.7 | 14.8 | 25.8 | 48.7 |

Reference

1. Yang, S. et al. Oxidation state investigation concerning liquid crystal alignment on polydimethylsiloxane layer by ion beam irradiation. *Liquid Crystals* **39**, 71-75 (2012).
2. Lin, P.-C. & Yang, S. Spontaneous formation of one-dimensional ripples in transit to highly ordered two dimensional herringbone structures through sequential and unequal biaxial mechanical stretching. *Appl. Phys. Lett.* **90**, 241903 (2007).
